# Delegates to the American Medical Association

**Published:** 1856-04

**Authors:** 


*Delegates to the American Medical Association.*

The following are the Delegates appointed by the Illinois State Medical Society, to attend the next annual meeting of the American Medical Association, viz: Drs. A. S. McArthur, F. A. McNeil, R. Rouse, C. N. Andrews, S. Y. Baldwin, E. Andrew, H. Noble, G. M. Morse, E. Goodbrake, H. A. Johnson, and Dr. Stearns.

